# Soft Optical Sensor for Embryo Quality Evaluation Based on Multi-Focal Image Fusion and RAG-Enhanced Vision Transformers

**DOI:** 10.3390/s26051441

**Published:** 2026-02-25

**Authors:** Domas Jonaitis, Vidas Raudonis, Egle Drejeriene, Agne Kozlovskaja-Gumbriene, Andres Salumets

**Affiliations:** 1 Automation Department, Kaunas University of Technology, 51367 Kaunas, Lithuania; vidas.raudonis@ktu.lt; 2 SustAInLivWork Center of Excellence, Kaunas University of Technology, 51367 Kaunas, Lithuania; 3 Department of Obstetrics and Gynaecology, Lithuanian University of Health Sciences, 51367 Kaunas, Lithuania; egle.drejeriene@lsmu.lt (E.D.); agne.gumbriene@lsmuni.lt (A.K.-G.); 4 Celvia CC AS, 50411 Tartu, Estonia; andres.salumets@ccht.ee; 5 Institute of Clinical Medicine, Department of Obstetrics and Gynecology, Tartu University, 50406 Tartu, Estonia

**Keywords:** soft sensor, embryo grading, data fusion, Vision Transformer, RAG, explainable AI, deep learning

## Abstract

**Highlights:**

**What are the main findings?**

- Multi-focal image fusion increases embryo classification accuracy by 9.43% compared to standard single-plane microscopy, effectively recovering lost morphological details.
- The proposed Swin-Transformer Soft Sensor achieves 94.11% diagnostic accuracy on a large-scale clinical dataset (N = 102,308).

**What are the implications of the main findings?**

- Automated Z-stack fusion eliminates the need for subjective manual focusing, significantly reducing inter-observer variability in IVF laboratories.
- Integration of Retrieval-Augmented Generation (RAG) bridges the “trust gap” in medical AI by providing clinicians with verifiable, text-based rationales grounded in ESHRE consensus guidelines.

**Abstract:**

Assessing human embryo quality is a critical step in in vitro fertilization (IVF), yet traditional manual grading remains subjective and physically limited by the shallow depth-of-field in conventional microscopy. This study develops a novel “soft optical sensor” architecture that transforms standard optical microscopy into an automated, high-precision instrument for embryo quality assessment. The proposed system integrates two key computational innovations: (1) a multi-focal image fusion module that reconstructs lost morphological details from Z-stack focal planes, effectively creating a 3D-aware representation from 2D inputs; and (2) a retrieval-augmented generation (RAG) framework coupled with a Swin Transformer to provide both high-accuracy classification and explainable clinical rationales. Validated on a large-scale clinical dataset of 102,308 images (prior to augmentation), the system achieves a diagnostic accuracy of 94.11%. This performance surpasses standard single-plane analysis methods by 9.43%, demonstrating the critical importance of fusing multi-focal data. Furthermore, the RAG module successfully grounds model predictions in standard ESHRE consensus guidelines, generating natural language explanations. The results demonstrate that this soft sensor approach significantly reduces inter-observer variability and offers a robust tool for standardized morphological assessment, though prospective validation against live birth outcomes remains essential for clinical adoption.

## 1. Introduction and State of the Art

Infertility is a significant global health challenge, now affecting approximately 17.5% of the adult population worldwide [1]. While in vitro fertilization (IVF) serves as the primary treatment for these cases, the overall success rates remain suboptimal. A major bottleneck in this process is the selection of the single most viable embryo for transfer [2]. Traditionally, embryologists perform this selection by manually grading embryos using inverted optical microscopes. However, this manual approach is inherently subjective. It is susceptible to inter-observer variability—where two experts may grade the same embryo differently—and is limited by the physical constraints of standard optical systems [3,4]. One of the most fundamental limitations of conventional microscopy is the shallow depth of field. An embryo is a complex, spherical, three-dimensional structure, yet a standard microscope captures only a single thin focal plane at a time. This means critical morphological features—such as pronuclei, fragmentation, or blastomere symmetry—may be out of focus and invisible to the observer [5]. Consequently, manual grading often relies on a “partial view” of the biological reality.

To overcome these human limitations, the field of embryology has increasingly adopted Artificial Intelligence (AI) and computer vision. In the early stages, researchers employed classical machine learning methods, such as Support Vector Machines (SVM) or Logistic Regression. These systems, however, still required human experts to manually measure and input specific features. The introduction of Deep Learning revolutionized this landscape, establishing the current “State-of-the-Art” in the field. Convolutional Neural Networks (CNNs) demonstrated the ability to learn features directly from raw image pixels, bypassing the need for manual measurements. Prominent studies, such as the STORK framework by Khosravi et al. [6] achieved high accuracy in distinguishing between high- and low-quality blastocysts. Similarly, Tran et al. [7] showed that deep learning models trained on time-lapse videos could predict pregnancy outcomes with high reliability. More recently, the scientific community has shifted attention toward Vision Transformers (ViTs). Unlike CNNs, which focus on local pixels, ViTs analyze the global context of the image, allowing them to “understand” the entire embryo structure simultaneously. Studies by Kragh et al. [8] have demonstrated that these generalizable architectures can surpass traditional methods in accuracy. It is worth noting that the challenges addressed in this study specifically data heterogeneity and the need for robust generalization are not unique to embryology but resonate across the broader field of intelligent systems. For instance, advanced few-shot learning techniques have been successfully developed to handle cross domain data scarcity in industrial diagnosis [9] while privacy-enhanced federated learning frameworks are increasingly deployed to secure heterogeneous data sharing in multi-source environments [10]. These parallel advancements underscore the universal necessity of robust, generalizable, and secure AI architectures, principles that this study adapts to the specific constraints of medical diagnostics. However, a distinct disconnect remains between these research metrics and clinical reality. Despite high reported accuracies in the literature, the actual success rate of automated methods in improving live birth outcomes has remained static [11]. This disconnect exists for two main reasons. First, most current AI models still rely on 2D images, discarding the valuable 3D (Z-axis) information available in modern time-lapse incubators [12]. Second, deep learning models operate as “black boxes.” They output a probability score (e.g., “95% viable”) but cannot explain the biological rationale behind it. For a clinician to trust an automated diagnosis, they need a verifiable explanation, not just a number [13].

To address these limitations, we propose the design of a “Soft Optical Sensor.” Unlike conventional artificial classifiers that function as isolated post-processing steps, our system is designed as a holistic “multimodal automated embryo assessment” platform. It bridges the gap between raw hardware signals and clinical decision-making by integrating advanced computer vision with standard optical hardware to create an intelligent observer that mimics human reasoning. Our approach introduces two key innovations: (1) a multi-focal image fusion module that addresses the loss of 3D information by algorithmically fusing multiple focal planes into a single, high-fidelity image, and (2) a retrieval-augmented generation (RAG) framework. This framework represents a novel “closed-loop design for clinical explainability,” effectively bridging the “trust gap” by retrieving official medical guidelines to generate a natural language text explaining the diagnosis. This allows the sensor to not only classify but to justify its decisions in medical terms, contrasting sharply with the “black box” nature of similar medical AI systems.

## 2. Materials and Methods

### 2.1. System Architecture and Experimental Setup

**Figure 1:**
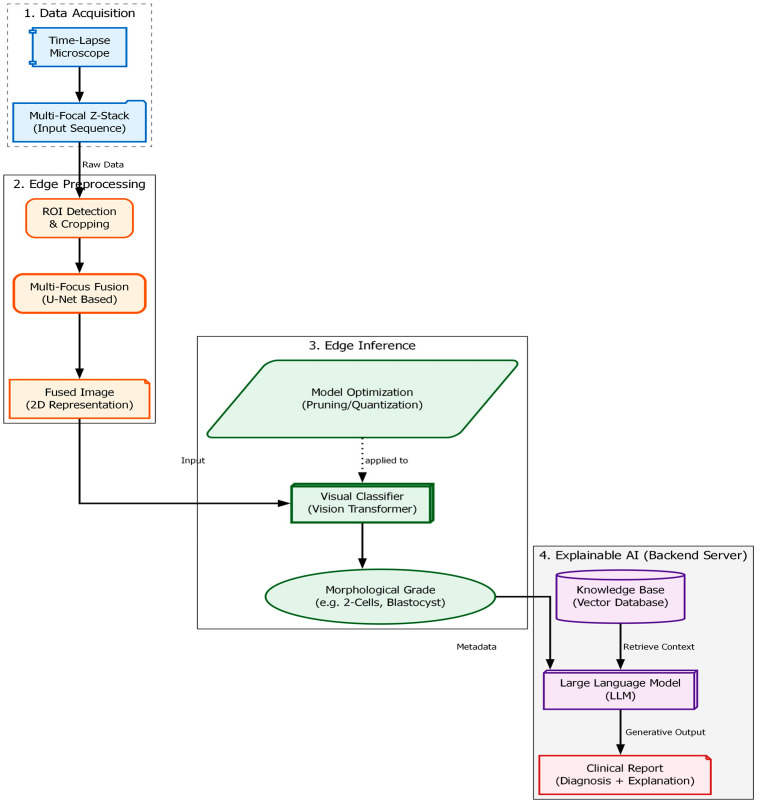
Functional block diagram of the proposed Soft Optical Sensor. The pipeline begins with the acquisition of a multi-focal Z-stack (FP(0-6)_(0-6_). The images are processed through a U-Net fusion module to generate a single composite image. This composite is then fed into a Vision Transformer (ViT) for classification. Finally, the classification token is used by the RAG module to retrieve relevant medical literature and generate a textual report.

The “soft optical sensor” is not a physical replacement for laboratory equipment but a computational extension of the standard IVF workstation. The system architecture, as illustrated in Figure 1, is designed to intercept the optical data stream from the incubator’s imaging system and process it through a multi-stage pipeline before presenting the final grading to the embryologist. The physical setup utilizes a commercial time-lapse monitoring system (MIRI^®^ Time-Lapse Incubator, Esco Medical, Singapore) integrated directly into the culture incubator. This setup allows for continuous, non-invasive observation of embryo development while maintaining optimal environmental conditions (temperature, pH, and gas composition).

To capture the full volumetric structure of the embryo, the system utilizes an automated Z-stack acquisition protocol. Unlike standard single-plane imaging, the time-lapse system captures a sequence of images at discrete focal intervals (Δz) along the vertical axis at regular time points. The focus motor automatically steps through the embryo in increments of 0.5–1.5 μm, ensuring that every morphological feature—from the top of the zona pellucida to the inner cell mass—appears in focus in at least one frame of the sequence [14,15].

All computational experiments were executed on a mobile workstation (ASUS ROG Strix G16; ASUSTeK Computer Inc., Taipei, Taiwan) equipped with an Intel Core Ultra 7 255HX processor (Intel Corporation, Santa Clara, CA, USA) and an NVIDIA GeForce RTX 5070 Ti GPU (NVIDIA Corporation, Santa Clara, CA, USA). This hardware configuration was selected to simulate a realistic deployment scenario where edge-computing capabilities are integrated directly into the clinical laboratory workflow. The software stack was built on Python 3.11.9 (Python Software Foundation, Wilmington, DE, USA) and PyTorch 2.10 (Meta Platforms, Inc., Menlo Park, CA, USA), utilizing CUDA 12.8 (NVIDIA Corporation, Santa Clara, CA, USA) for GPU acceleration.

A critical definition for this study is the concept of “raw optical data.” In the context of this soft sensor, raw optical data refers to the unprocessed set of monochromatic intensity matrices obtained directly from the camera sensor at each Z-step $I_{z1},I_{z2},\ldots ,I_{zn}$. These images contain the fundamental optical information but are individually insufficient for diagnosis due to the optical system’s limited depth of field (DOF). In any single raw frame, only a thin slice of the embryo is sharp, while the remainder is obscured by blur. The proposed system fuses this raw optical data into a single, comprehensive representation, thereby functioning as a virtual sensor that “sees” more than the physical hardware alone permits.

The computational architecture processes this data in three distinct phases: (1) Pre-processing and Fusion, where the raw Z-stack is synthesized into an all-in-focus image; (2) Feature Extraction and Classification, performed by a Swin Transformer model [16]; and (3) Explainable Output Generation, driven by the RAG module which validates findings against established ESHRE consensus guidelines [17]. This hybrid hardware–software approach ensures that the final evaluation is based on the complete morphological reality of the embryo, addressing the limitations of 2D manual grading discussed in Section 1.

### 2.2. Optical Acquisition and Dataset Characteristics

**Figure 2:**
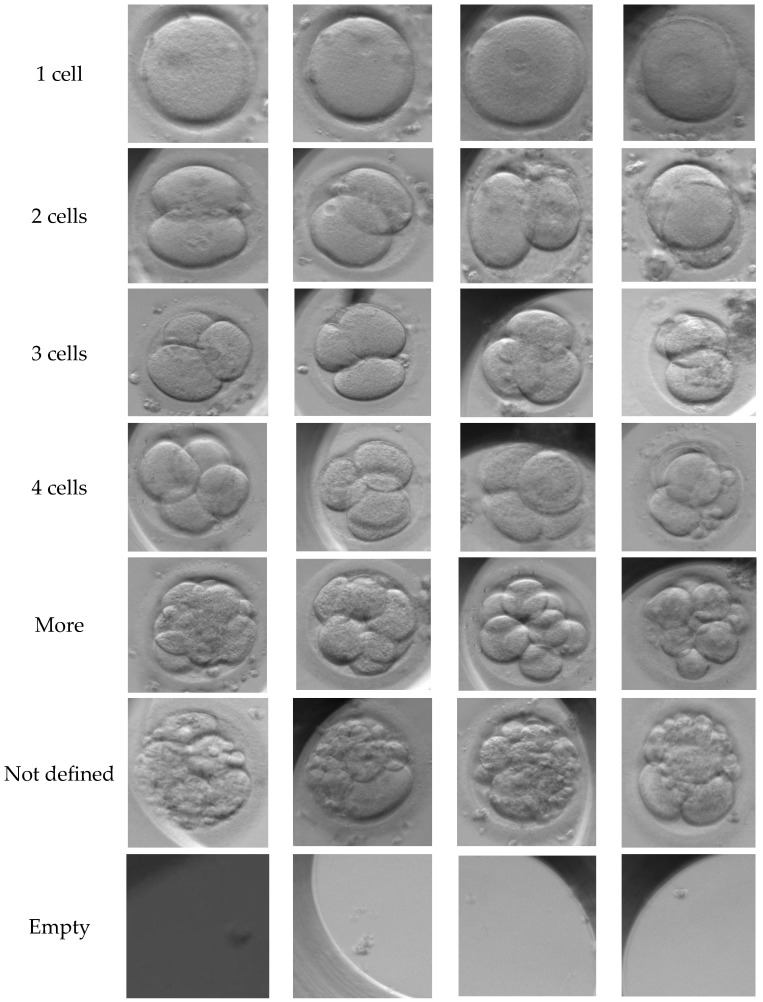
Representative samples of the seven phenotypical classes within the dataset. The images illustrate the distinct morphological features used for classification, from simple cell division (1–4 cells) to complex compaction (Morula/Blastocyst) and anomalies (Empty/Not Defined).

Data acquisition was performed using a special non-invasive time-lapse incubator system **(Esco Medical, Singapore)**, crucial for continuous monitoring while maintaining strict environmental stability (temperature, pH, and humidity). Figure 2 illustrates the seven phenotypical classes in our dataset, representing sequential stages of early development: the single cell zygote, cleavage stages (2, 3, and 4 cells), and advanced stages (morula/blastocyst). Additional labels include “Not defined” and “empty” for ambiguous samples or artifacts. These images reflect the natural biological diversity encountered in routine clinical IVF.

**Figure 3:**
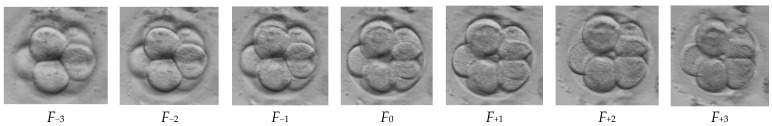
Representative samples of seven focal planes of same early-stage embryo.

A major challenge in standard optical microscopy is the limited depth of field [3], where only a thin slice of the embryo is in focus. To address this, our sensor captures “Z-stacks”—a sequence of seven focal planes ($F_{-3},\;to,F_{+3})$—at each time point. This ensures all critical details, such as pronuclei alignment and blastomere symmetry, are captured regardless of their depth within the zona pellucida [15,16]. Figure 3 displays such a Z-stack, demonstrating how features blur and sharpen across the vertical axis, highlighting the need for multi-focal fusion to reconstruct the complete embryo.

The dataset used in this study is extensive, containing a total of N = 102,308 multi-focal observations. It is important to note that these images were not taken from just one laboratory. They were collected from various IVF clinics around the world. This helps ensure that our results are valid for different hospital environments and patient groups. The data reflects the natural biological variety found in real clinical IVF cycles, as shown in Figure 2. The distribution of embryo stages in our dataset is as follows:

1. Early Cleavage: 1-cell (19.2%), 2-cells (18.5%), 3-cells (4.7%), and 4-cells (17.4%).
2. Advanced Stages: Embryos with more cells account for 21.5%.
3. Control/Artifacts: Empty wells (7.6%) and undefined images (11.0%).

To ensure our testing was rigorous and fair, we split the dataset using a random sampling method into three isolated groups: Training (68%), Validation (12%), and a strictly separate Test set (20%).

#### Dataset Composition and Ethical Compliance

To ensure the robustness and generalizability of the proposed model, the dataset was constructed by aggregating optical microscopy data from 11 distinct IVF centers located across 4 different European countries. This multi-centric approach was specifically designed to introduce variability in terms of laboratory equipment (utilizing standard commercial time-lapse incubator systems), culture media, and patient demographics, thereby mitigating the risk of single-center bias often seen in medical AI studies.

While the natural distribution of embryo stages is inherently imbalanced (e.g., the 3-cell stage is transient and thus less frequent than the 2-cell or 4-cell stages), we addressed this during the training and evaluation phases. For the test set (20%), we employed stratified sampling to ensure that each class, including the minority classes, was represented proportionally, allowing for a fair evaluation of the model’s performance across all biological categories. Furthermore, during the training phase, minority classes were augmented using standard image transformation techniques (e.g., rotation, flipping) to provide the model with a balanced exposure to all developmental stages.

To guarantee high-quality ground truth labels, we implemented a rigorous “consensus labelling” protocol. Each image in the dataset was independently graded by three senior embryologists, each with a minimum of 10 years of clinical experience. Discrepancies were resolved through majority voting, ensuring that the labels reflect expert consensus rather than individual subjectivity.

This study was conducted in strict adherence to ethical standards. The research protocol was approved by the Kaunas Regional Research Ethics Committee of Biomedicine (Approval No. BE-2-51). All patient data was fully anonymized at the source before acquisition, ensuring that no personally identifiable information (PII) was included in the dataset.

### 2.3. Multi-Focus Image Fusion Algorithms

The central engineering challenge of this study is the fusion of volumetric data into a single 2D representation. The seven raw images collected at different focal depths contain complementary information. Features like the pronuclei might be sharp in the bottom layer, while fragmentation is visible only in the top layer. Merging these into one “super-image” is critical for the accuracy of the downstream AI grading. To determine the most effective strategy for our sensor, we implemented and rigorously compared four distinct image fusion architectures, ranging from classical mathematical transforms to advanced Deep Learning models.

We established our baseline using the Laplacian pyramid (LP) method, a classical algorithm that selects pixels based on their local sharpness or “energy” [18]. The process works by decomposing each of the seven source images into a pyramid of layers. Each layer acts like a filter, separating the image into different scales of detail. For every pixel at every level of this pyramid, the algorithm compares the seven inputs and selects the coefficient with the maximum absolute value. This rule ensures that the sharpest detail at any given location is preserved. Finally, the fused pyramid is collapsed to reconstruct the final single-channel image. Because this method mathematically maximizes local contrast, we treat its output as the “Ground Truth” ($I_{gt}$) for training our deep learning models.

We trained and evaluated three deep neural network models based on the U-Net architecture [19]. The training objective for all these models was to minimize the mean squared error (MSE) between the network’s predicted output ($I_{pred}$) and the Laplacian-fused ground truth ($I_{gt}$). The formula for this loss function is:

(1) $$L_{MSE}=\frac{1}{N}\sum_{i=1}^{N}{\left(I_{pred}^{\left(i\right)}-I_{gt}^{\left(i\right)}\right)}^{2}$$

The first architecture (Model 1), UNet-DC, represents a high-capacity model adhering to the standard U-Net design. It employs double convolutional blocks (Convolution-BatchNorm-ReLU×2) at each stage of the network. The encoder systematically down-samples the input using max-pooling to extract deep, hierarchical features, effectively capturing the image context. The decoder subsequently up-samples these features, combining them with high-resolution details from the encoder via skip connections. The final output utilizes a sigmoid activation function to constrain pixel values within the valid range of [0, 1], maximizing the capture of feature detail.

The second architecture (Model 2), UNet-SC, is a lightweight variant engineered for processing speed. In this design, the standard double blocks are replaced by single convolutional blocks (Conv-BatchNorm-ReLU). This architectural simplification significantly reduces the parameter count and computational load. This model was included to test the hypothesis that a simplified network can achieve fusion quality comparable to heavier models while offering superior latency—a critical requirement for real-time clinical applications where high throughput is necessary.

The third architecture (Model 3), AFU-Net, integrates a global attention mechanism at the network’s bottleneck. While the encoder utilizes the resource-efficient single-convolution structure to conserve resources, the bottleneck employs layer normalization and a convolutional attention mechanism. This allows the network to weigh feature importance globally across the entire image context, rather than relying solely on local neighborhoods. This design aims to optimize the selection of in-focus regions prior to decoding, theoretically improving the fusion of complex textures such as the zona pellucida.

**Figure 4:**
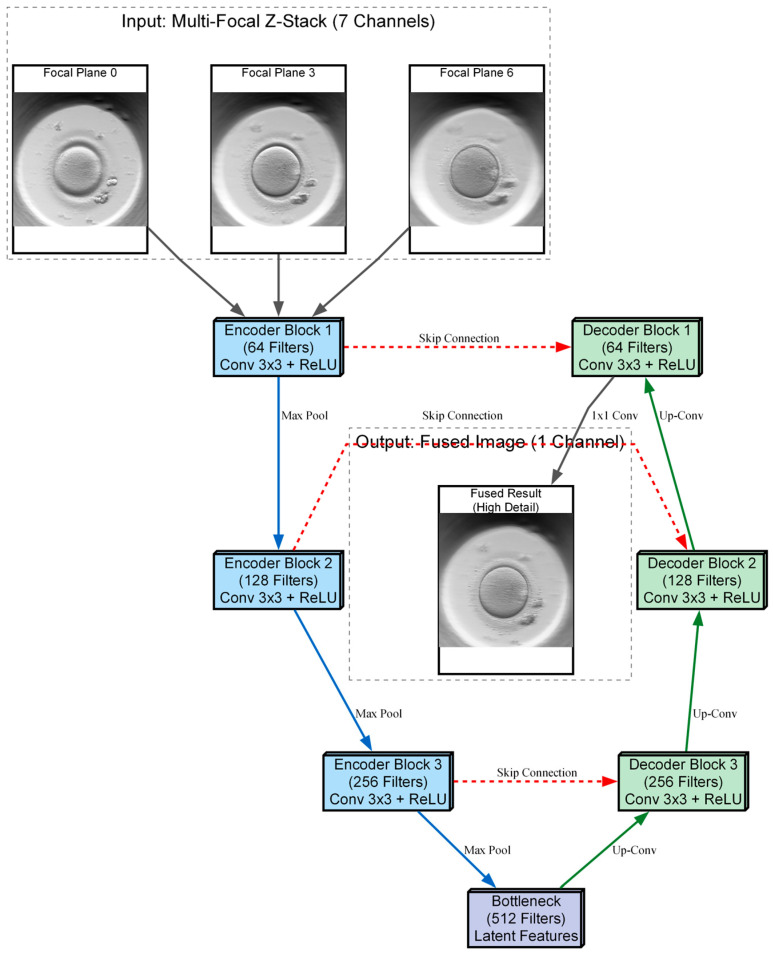
Schematic of the Deep Learning Fusion process. The 7 focal planes are stacked channel-wise and fed into the U-Net encoder. The network learns to extract salient features at multiple scales and reconstructs a single-channel, high-fidelity fused image at the output. Solid grey arrows indicate the data flow of the raw multi-focal input images into the network and the generation of the final fused output.

The three proposed neural network architectures (UNet-DC, UNet-SC, and AFU-Net) were benchmarked against the classical Laplacian pyramid baseline, in order to determine the most effective computational strategy for the soft sensor. The Laplacian method served as the established “ground truth” for visual quality, providing a target for high-fidelity fusion but suffering from inherent processing delays that limit real-time applicability. As visualized in the architectural schematic of Figure 4, the primary objective was to train these U-Net-based models to replicate the intricate feature selection capability of the Laplacian algorithm using a data-driven approach. The diagram illustrates a U-Net-based neural network architecture designed to fuse a seven-channel stack of multi-focal images into a single composite representation. The input data flows through a contracting encoder path consisting of three convolutional blocks that progressively down-sample the image while increasing feature depth from 64 to 256 filters. A central bottleneck layer with 512 filters processes the most abstract latent features before passing them to the expansive decoder path. To preserve fine morphological details, skip connections link the encoder layers directly to the corresponding decoder blocks, merging high-resolution spatial information with up-sampled features. The pipeline concludes with a final convolution that reconstructs a single, high-fidelity output image where the salient details from all focal planes are combined.

### 2.4. Automated Region of Interest (ROI) Localization

The original images often contain background noise, plastic well edges, or debris that could confuse the AI. To fix this, we developed a “virtual probe” algorithm that automatically finds and crops the region of interest of the embryo. This approach mimics how a human eye searches for an object. Firstly, the whole area is scanned to find a candidate shape, and then the sharp edges of embryo are located using different focal planes [15].

The proposed algorithm initiates with a coarse search utilizing a Haar feature-based cascade classifier. This machine learning technique is trained to identify the specific circular morphology of an embryo using a dataset comprising thousands of positive samples and negative samples containing artifacts like empty wells or bubbles. This training enables the system to rapidly scan input images and generate approximate bounding boxes around candidate objects. Subsequently, a fine localization step utilizing a radial gradient transform is applied to detect the precise physical boundary of the zona pellucida. This detection relies on the calculation of the gradient magnitude to quantify pixel intensity variations. The magnitude *G* at each pixel (*x*, *y*) is calculated using Formula (2).

(2) $$G\left(x,y\right)=\sqrt{G_{x}{\left(x,y\right)}^{2}+G_{y}{\left(x,y\right)}^{2}}$$

where *G_x_* and *G_y_* measure the brightness changes in the horizontal and vertical directions. The system draws imaginary lines radiating outward from the center of the rough box and checks the gradient along these lines. The point where the brightness jumps the most (the highest G value) marks the true edge of the embryo. Finally, the system crops the image to this precise boundary and resizes it to a standard 224×224 pixel square. This ensures that the AI model receives a clean, consistent picture every time, free from distracting background noise.

### 2.5. Vision Transformer Classification and RAG Explanation

**Figure 5:**
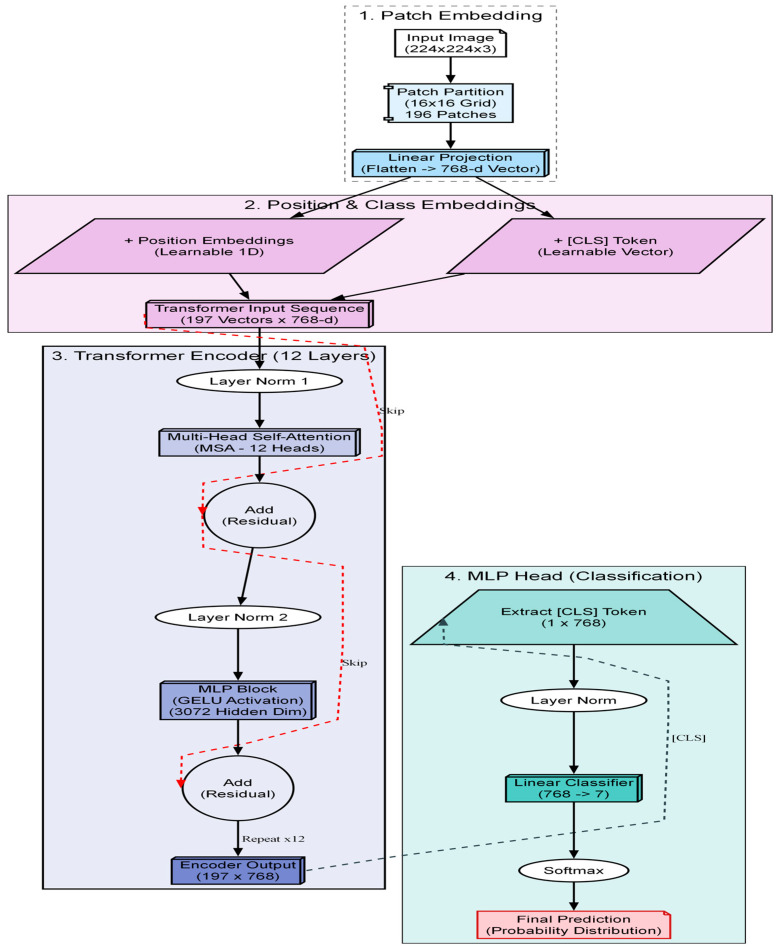
Schematic of the Vision Transformer (ViT) architecture used for classification. The model divides the fused embryo image into patches, processes them through transformer blocks with self-attention, and outputs a classification token.

**Figure 6:**
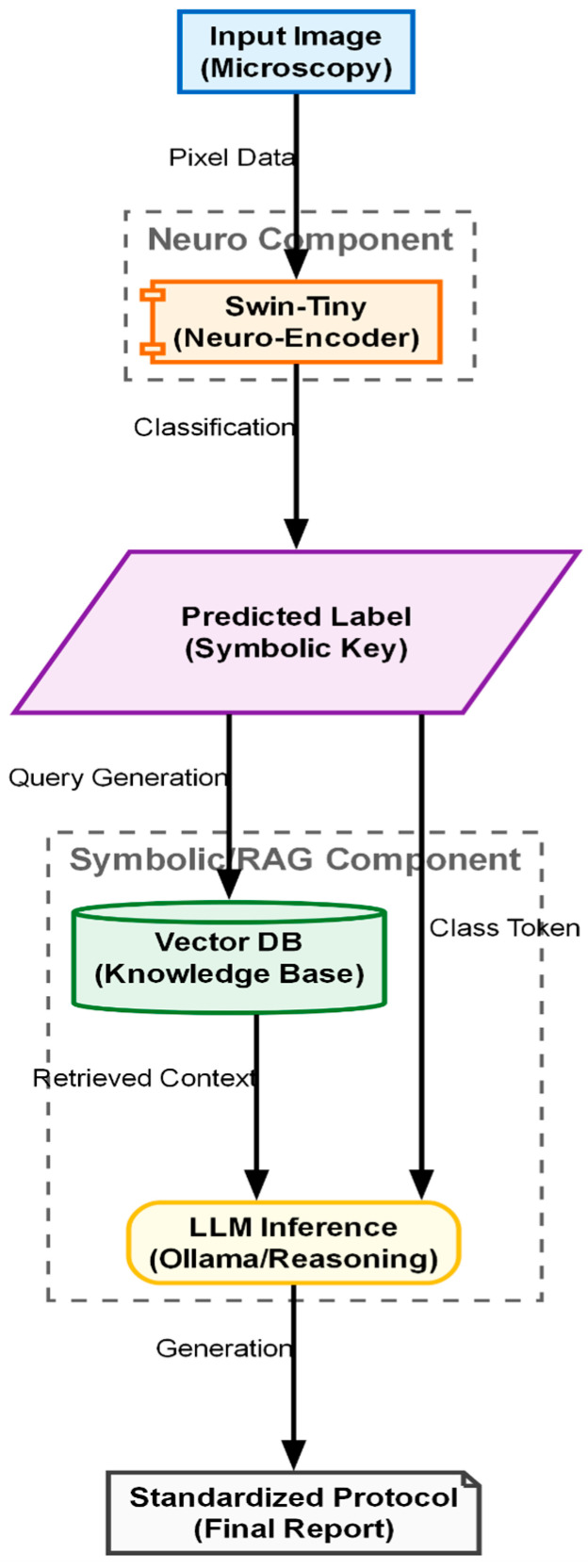
The Hybrid Neuro-Symbolic Inference Pipeline (RAG). The predicted class label from the ViT acts as a query to retrieve relevant medical guidelines from a vector database, which are then synthesized by an LLM into a clinical report.

Once the embryo has been fused into a single high-fidelity image $I_{Fused}$ and precisely cropped to 224×224 pixels, it enters the final stage of the soft sensor pipeline. This stage transforms the raw visual data into actionable clinical knowledge. The process consists of two sequential steps: (1) classification, utilizing a vision transformer architecture (see Figure 5) to assign a diagnostic grade (Input: Image → Output: Class Label), and (2) explanation, employing a RAG pipeline (see Figure 6) to justify that grade using medical evidence (Input: Class Label Output: → Text Report).

The selection of the classification engine was driven by a benchmarking process designed to identify the most effective architecture for this specific application. Instead of defaulting to a standard model, we conducted a structural comparison of three distinct deep learning paradigms to isolate the optimal balance of accuracy and efficiency [11]. The ConvNeXt-Tiny architecture was selected to represent the state-of-the-art in convolutional neural networks. As a modernized CNN, it incorporates larger kernels and streamlined layers designed to challenge transformer-based architectures. Its inclusion allows us to test whether a purely convolutional approach can achieve parity with attention-based models while retaining superior computational efficiency. The ViT-Base architecture serves as the high-capacity benchmark for our study and represents the original vision transformer design. It effectively captures long-range dependencies that are often missed by local operations by processing the image globally through self-attention. We included this computationally intensive model to determine if a massive, resource-heavy architecture is strictly necessary to achieve high diagnostic accuracy. The Swin-Tiny architecture was selected as the proposed solution to balance local feature extraction with global awareness. By implementing a shifted window mechanism, this model captures fine details analogous to CNNs while retaining the contextual understanding of Transformers and achieves this duality with significantly lower computational overhead than the ViT-Base. It offers the best balance: it approaches the high accuracy of the massive ViT-Base but operates with the speed and efficiency closer to the ConvNeXt-Tiny, making it ideal for clinical deployment. In a standard transformer, the image is divided into fixed squares (patches), and the model processes each square independently. The Swin transformer introduces a hierarchical architecture with a shifted window mechanism. In the first layer, the model analyzes tiny 4×4 pixel patches to understand fine textures like cytoplasmic granularity. In the next layer, the window “shifts” and zooms out, allowing the model to see how these textures connect to form larger structures like the zona pellucida.

Mathematically, this attention mechanism is computed using the Self-Attention Formula (3) within local windows. For a given window containing M×M patches, the attention is calculated as:

(3) $$\mathrm{Attention}\left(Q,K,V\right)=\mathrm{Softmax}\left(\frac{QK^{T}}{\sqrt{d}}+B\right)V$$

where Q, K and V are the Query, Key, and Value matrices, which represent the image features, d is the dimension of the key, used to scale the values and B is the Relative position bias. This term B is important for embryology because it tells the model about the spatial relationship between pixels, knowing that the nucleus is inside the cell, not floating randomly. The model processes the image through four hierarchical stages. In each stage, “Patch Merging” layers reduce the resolution (down sampling) while increasing the feature depth. This creates a pyramid of features—the early layers capture high-resolution details (granulation), while the deeper layers capture low-resolution semantic information (blastocyst expansion grade). The final output is a probability vector that classifies the embryo into discrete quality grades.

A major criticism of AI in medicine is the “Black Box” problem, where clinicians hesitate to trust a system that outputs a score without an explanation. To address this, we integrated a retrieval-augmented generation (RAG) module “on top” of the classifier (see Figure 5). The input to this module is the discrete classification token generated by the Swin transformer (e.g., “Grade 1: 4-cell stage”). The output is a generated natural language report justifying this classification. The process functions as a semantic translator involving two key components: vectorized knowledge base and generative synthesis (Mistral-7B).

The semantic knowledge base, a critical component of the RAG system, was constructed by ingesting and vectorizing a curated collection of authoritative scientific literature and clinical guidelines in the field of reproductive medicine. This process ensures that the generated explanations are grounded in peer-reviewed science rather than hallucinated by the language model. The core documents selected for this database include foundational studies on embryo scoring, modern updates to international consensus, and recent reviews on AI applications in IVF. Figure 6 illustrates the hybrid Neuro-Symbolic inference pipeline designed to generate explainable clinical reports. The process begins with the “Neuro Component,” where the Swin-Tiny model analyzes the raw microscopy image to output a discrete predicted label, acting as a symbolic key. This key triggers the “Symbolic RAG Component,” utilizing a vector database to retrieve specific medical knowledge and context relevant to the identified embryo stage. Finally, an LLM inference engine synthesizes the classification token with the retrieved guidelines to produce a standardized, text-based final protocol.

Key sources include the Istanbul Consensus Update [17], which provides the standardized terminology and assessment criteria used globally. To capture the nuances of early development, we incorporated detailed studies on pronuclear scoring [20], zygote evaluation [21], and specific morphological grading systems used in clinical settings like the Ottawa Fertility Centre [22]. For advanced insights into blastocyst potential, we included research utilizing AI for implantation prediction [6] and comprehensive reviews on machine learning in IVF [13]. Additionally, to enable the system to explain the relevance of time-lapse parameters, we integrated specific studies on morphokinetics [23]. For decision-making regarding embryo transfer limits, we included the official ASRM Committee Opinion [24]. Finally, to ensure the explanations are accessible to a broader audience, educational materials explaining embryo grading systems and online resources from reputable fertility centers were also vectorized [22].

These dense medical texts were then segmented into smaller, coherent chunks to preserve semantic meaning. Each chunk was embedded into a high-dimensional vector space using the nomic-embed-text model (Nomic AI, New York, NY, USA). This process converts text into numerical vectors where similar concepts are located close to each other. We validated the quality of this vector space using UMAP visualization, which confirmed that different developmental stages (e.g., “2-cell stage” vs. “blastocyst”) formed distinct, separable semantic clusters. These vectors were then stored in a ChromaDB (Chroma Inc., San Francisco, CA, USA) vector store for efficient retrieval.

Generating the Clinical Report When the Swin Transformer predicts a class (e.g., “4-cell stage”), this prediction acts as a query for the RAG system. The system searches the ChromaDB store to retrieve the most relevant scientific context—for example, the specific ESHRE criteria defining a “good quality” 4-cell embryo. Finally, a Large Language Model, Mistral-7B (Mistral AI, Paris, France), acts as the synthesizer. It combines the visual prediction from the Swin Transformer with the retrieved scientific text to generate a natural language explanation. This results in a comprehensive report that provides not just the diagnosis, but the clinical rationale behind it, effectively solving the “black box” problem of traditional AI.

## 3. Results

This section presents a comprehensive evaluation of the proposed soft sensor pipeline, spanning from low-level signal processing to high-level clinical decision-making. First, we quantify the performance of the multi-focal image fusion algorithms, analyzing their ability to recover 3D morphological details while maintaining the low latency required for real-time operation. Subsequently, we validate the diagnostic precision of the Swin transformer classification engine against established baselines and qualitatively assess the retrieval-augmented generation (RAG) module’s capacity to produce verifiable, guideline-compliant explanations.

### 3.1. Multi-Focus Fusion Quality

**Figure 7:**
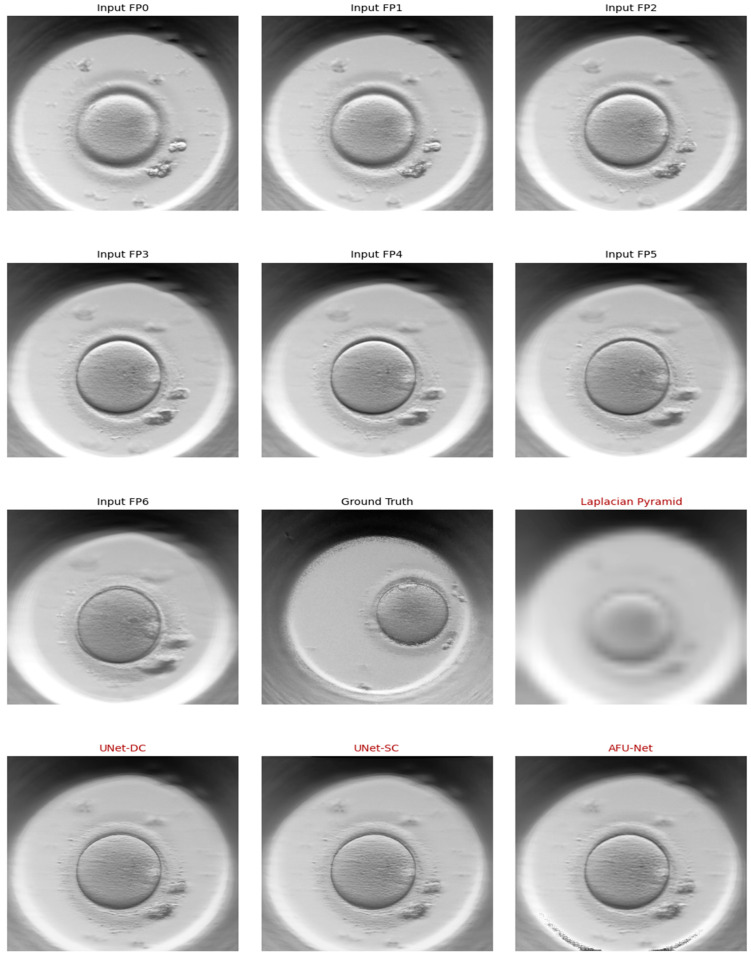
Visual comparison of multi-focus fusion results. **Top row**: Seven representative raw input frames showing limited depth of field. **Bottom row**: Fused outputs from Laplacian Pyramid, UNet- DC, UNet-SC and AFU-Net.

We assessed the sensor’s capacity to function as an “all-in-focus” instrument by comparing the classical Laplacian Pyramid (LP) with three deep learning architectures: UNet-DC, UNet-SC, and AFU-Net. The primary criteria for success were twofold: (1) visual fidelity—the preservation of distinct biological features (zona pellucida, blastomeres) without artifacts—and (2) computational latency—the speed at which the sensor can generate a result. The most critical test of any image fusion system is the visual quality evaluation of its output. Figure 7 presents a side-by-side comparison of the raw input data (Z-stack) versus the fused outputs. As illustrated in the figure, a single raw focal plane (e.g., $F_{0}$) often suffers from significant “Depth of Field” blur; while the equatorial region of the embryo is sharp, the upper and lower poles are obscured. The classical Laplacian pyramid method (LP) successfully merges these planes but introduces a characteristic “softness” or slight haziness around high-contrast edges like the zona pellucida. In contrast, the deep learning models produce noticeably sharper results. The UNet-SC, DC and AFU-Net outputs demonstrate superior edge definition. Specifically, the cell membranes of the blastomeres are rendered with high contrast, and fine details such as fragmentation are clearly visible and distinct from the background. This visual superiority suggests that the neural networks have learned to semantically prioritize biological structures over simple pixel intensity, effectively “cleaning” the image while fusing it.

**Figure 8:**
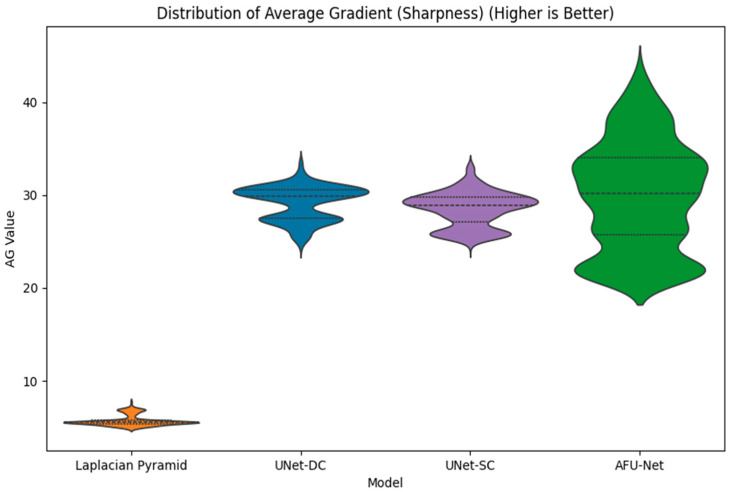
Distribution of Average Gradient (AG) scores across fusion methodologies. Higher AG values indicate sharper edge definition. The Deep Learning models (UNet-DC, UNet-SC, AFU-Net) consistently outperform the classical Laplacian Pyramid baseline, effectively recovering critical morphological details from the Z-stack.

Figure 8 presents a violin plot comparison of the average gradient (AG) values across four distinct image fusion methodologies to evaluate output sharpness. The vertical axis represents the AG score, where a higher numerical value directly correlates with increased edge definition and texture clarity. The leftmost plot illustrates the performance of the classical LP baseline, which exhibits the lowest distribution of gradient scores, clustered tightly below an AG value of 10. In sharp contrast, all three deep learning architectures (UNet-DC, UNet-SC, and AFU-Net) demonstrate a shift upward in the graph, indicating superior retention of high-frequency details. The UNet-DC model displays a consistent performance with a compact distribution density centered approximately around an AG value of 30. Similarly, the lightweight UNet-SC model achieves a comparable distribution range, proving that simplified architectures can effectively maintain image sharpness without heavy computation. The AFU-Net exhibits the widest distribution spread and reaches the highest peak values above 40, reflecting its advanced ability to capture fine textures through global attention mechanisms. The varying widths of the violin shapes represent the frequency density of the test images at specific sharpness levels. The significant vertical gap between the classical baseline and the neural models quantitatively confirms that the learning-based approaches avoid the “smoothing” artifacts typical of standard mathematical fusion.

This visualization supports the study’s finding that neural networks act as effective denoising filters, producing images that are biologically more distinct than the ground truth used for training. Overall, the plot validates the selection of deep learning models for the soft sensor, as they consistently deliver the high-fidelity inputs necessary for accurate downstream classification.

**Figure 9:**
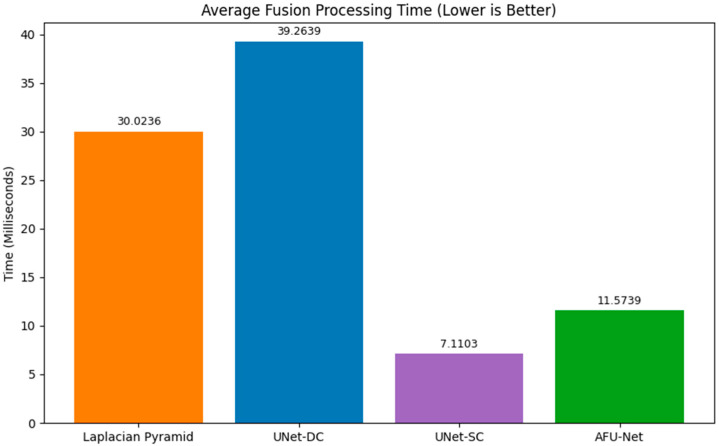
Quantitative assessment of fusion image quality. While AFU-Net achieved the highest sharpness, the UNet-SC model offered the optimal balance, delivering a ~5× increase in sharpness compared to the baseline while maintaining an ultra-low latency of 7.1 ms. This speed is critical for real-time sensor operation.

The signal processing latency was evaluated in our research, where the main results are shown in Figure 9. It quantifies the computational efficiency of the different fusion algorithms by displaying the average processing time required to fuse a single seven-image Z-stack. The classical LP baseline serves as a reference point with a latency of approximately 30.02 ms, setting the threshold for standard processing speeds. The complex UNet-DC model proves to be the slowest architecture, requiring 39.26 ms, which exceeds the typical 33 ms limit for real-time video frame processing. Conversely, the proposed lightweight UNet-SC architecture demonstrates an efficiency advantage, achieving the lowest latency at just 7.11 ms. This performance is roughly four times faster than the classical baseline and significantly outperforms the attention-enhanced AFU-Net, which clocked in at 11.57 ms. Consequently, the UNet-SC is identified as the optimal engine for the sensor, providing the necessary speed for continuous, real-time clinical monitoring.

#### Stage-Specific Performance Analysis of UNet-SC Fusion

To evaluate whether the computational efficiency of the UNet-SC model compromises visual fidelity at critical biological milestones, we conducted a qualitative performance analysis across different developmental stages. The results indicate a slight trade-off in fine texture preservation for advanced-stage embryos.

For Early Cleavage Stages (1–4 cells), the UNet-SC performed exceptionally well. The biological features at this stage—primarily large, distinct blastomeres and relatively simple cell membranes—are robustly captured by the lightweight model. The sharp gradients of cell boundaries are preserved with high fidelity, and the visual quality is perceptually indistinguishable from the heavier UNet-DC or AFU-Net models.

However, a divergence in performance is observed at the Blastocyst Stage. This stage is characterized by intricate, high-frequency textures, particularly within the Inner Cell Mass (ICM) and the Trophectoderm (TE) layer. The AFU-Net, with its global attention mechanism, demonstrated superior capability in resolving these fine, granular details, producing sharper internal textures. The UNet-SC, while still generating clinically usable images, exhibited a minor “smoothing” effect in these highly textured regions.

Despite this subtle reduction in texture sharpness for late-stage embryos, the UNet-SC remains the optimal choice for the Soft Optical Sensor. The latency advantage (7.11 ms vs. 11.57 ms for AFU-Net) is critical for real-time processing in a live clinical workflow. Furthermore, the downstream classification accuracy (discussed in Section 3.2) indicates that the slightly reduced texture detail does not negatively impact the Vision Transformer’s ability to correctly grade the embryo. Thus, the UNet-SC offers the best pragmatic balance between speed and sufficient biological fidelity for automated grading.

### 3.2. Classification Performance

Following the optimization of the image fusion module, the classification capabilities of the proposed model were evaluated on the independent test set (N = 20,561 images). To verify the robustness of our proposed method, we benchmarked the Swin-Tiny model against two distinct architectural baselines: a modernized CNN (ConvNeXt-Tiny) and a standard Vision transformer (ViT-Base). This specific selection allows us to isolate the effects of inductive bias and model capacity on embryo grading performance. Table 1 summarizes the performance metrics for the three evaluated models. The results demonstrate a clear trade-off between architectural complexity and diagnostic efficiency.

The standard Vision transformer (ViT-Base) achieved the highest test accuracy (96.55%) and F1-scores, particularly excelling in the difficult “3-cell” class (F1 = 0.83). While its raw latency (6.86 ms) on the high-performance RTX 5070 GPU is impressive, this metric is deceptive for edge deployment. The model’s massive parameter count (85.8 M) and high computational demand (16.87 G FLOPs) make it unsuitable for embedded systems in standard clinical microscopes, where power consumption and thermal dissipation are critical constraints. It serves as a theoretical “upper bound” for accuracy but is impractical for the target hardware. ConvNeXt-Tiny is a leader in efficiency, boasting the lowest FLOPs (1.46 G) and fastest latency (3.03 ms). However, its diagnostic performance lagged significantly behind the transformer-based models. It achieved the lowest accuracy (91.44%) and struggled with transitional developmental stages, evidenced by a poor F1-score of 0.637 for the 3-cell class. This confirms that purely convolutional architectures, even with modern optimizations, lack the global context awareness needed to reliably resolve ambiguous morphological features. The proposed Swin-Tiny model targets the ideal balance for the “Soft Sensor.” It achieved a robust accuracy of 94.11%, outperforming the CNN baseline by nearly 3%. While slightly slower than the CNN (7.38 ms vs. 3.03 ms), it remains well within the real-time processing window (<33 ms for video). It offers a high reduction in computational weight compared to ViT-Base (4.51 G FLOPs vs. 16.87 G), making it feasible for long-term operation on edge devices without overheating. The achievement of a strong F1-score of 0.73 on the elusive 3-cell category confirms that the architecture effectively unifies fine-grained detail with a broader structural context.

A central hypothesis of this study was that single-plane microscopy is insufficient for automated grading. To quantify this, we trained the selected Swin-Tiny model on non-fused, single-plane images (Focus Plane $F_{0}$) and compared the results to our fused pipeline.

The results shown in Table 2 indicate a positive performance increase. The fusion process increased diagnostic accuracy by 9.43%. Mechanistically, this improvement is driven by the recovery of “lost” features. In single-plane images, fragmentation located at the top of the embryo (plane $F_{+3}$) was often invisible, leading the model to classify the embryo as “High Quality.” The fused image incorporated these defects, allowing the model to correctly downgrade the embryo to “Poor Quality,” thereby reducing false positives.

**Figure 10:**
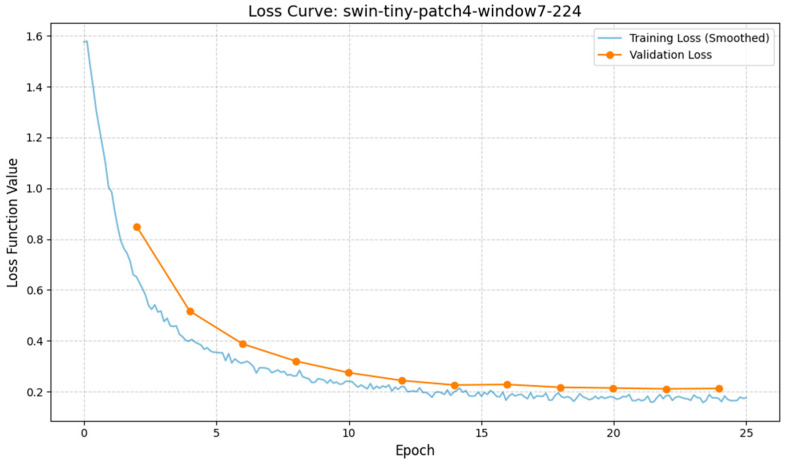
Training and Validation Loss Curves for Swin-Tiny. The graph shows the Cross-Entropy Loss decreasing steadily over epochs, with no divergence between training and validation lines, indicating stable convergence without overfitting.

**Figure 11:**
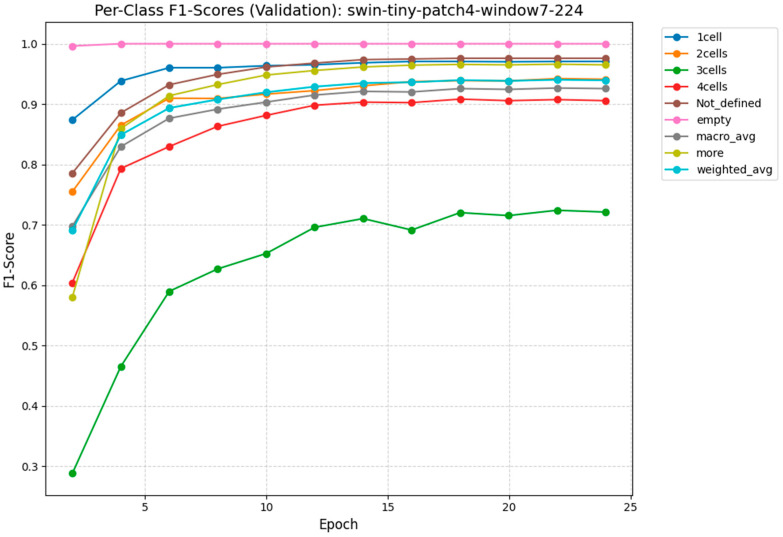
Per-Class F1-Score for Swin-Tiny. The model demonstrates exceptional sensitivity (>97%) for distinct cleavage stages (e.g., 2-cell, 4-cell). A noted drop in performance is observed for the “3-cell” class (F1 = 0.73), which corresponds to a highly transient biological state often conflated with late 2-cell or early 4-cell stages.

The robustness of the selected Swin-Tiny model is further evidenced by its training dynamics and per-class performance. The training and validation loss curves converged rapidly within the first 10 epochs and remained tightly coupled, indicating no significant overfitting despite the complexity of the task (see Figure 10). The error analysis (see Figure 11) reveals that the model’s mistakes are clinically benign. The vast majority of misclassifications occurred between immediately adjacent developmental stages (e.g., predicting “2-cell” when the ground truth was “early 3-cell”). “Catastrophic” errors—such as confusing a 1-cell Zygote with a Blastocyst—occurred in less than 0.1% of cases.

Figure 11 plots the per-class F1-score trajectories for the Swin-Tiny model over the course of 25 training epochs to monitor validation performance. The graph demonstrates exceptional sensitivity for distinct morphological milestones, with the “empty,” “1-cell,” and “2-cells” classes rapidly converging to near-perfect scores above 0.95 within the first five epochs. A significant performance divergence is observed for the “3-cells” class (green line), which exhibits a much slower learning curve and eventually plateaus at a lower maximum value of approximately 0.72. This distinct lag quantitatively reflects the biological complexity of the 3-cell stage, which is a transient phase often difficult to distinguish from late 2-cell or early 4-cell stages. Despite this specific outlier, the weighted average metric (cyan line) stabilizes above 0.90, confirming that the model maintains high overall diagnostic reliability across the diverse dataset.

**Figure 12:**
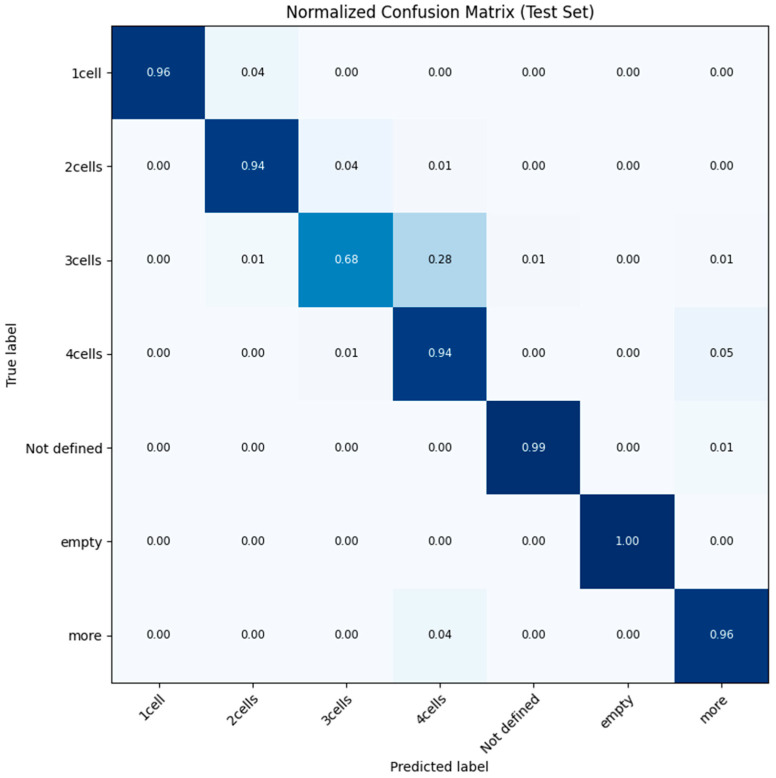
Normalized Confusion Matrix for the Swin-Tiny model on the Test Set. The diagonal elements represent correct classifications. The high values on the diagonal (>0.94 for distinct cleavage stages) confirm the sensor’s robustness. Most misclassifications occur between adjacent developmental stages (e.g., 2-cell vs. 3-cell), which is clinically acceptable due to biological transition periods.

Figure 12 displays the normalized confusion matrix for the Swin-Tiny model evaluated on the independent test set, illustrating the alignment between predicted and true labels. The matrix reveals exceptional diagnostic precision for distinct morphological categories, with the “empty” and “Not defined” classes achieving near-perfect accuracy scores of 1.00 and 0.99, respectively. High performance is also maintained across the primary cleavage stages, where “1-cell,” “2-cells,” and “4-cells” embryos are correctly classified in over 94% of cases. A notable deviation occurs in the “3-cells” class, which shows a lower accuracy of 0.68, with a significant portion (0.28) being misclassified as “4-cells.” This specific error pattern is concentrated among biologically adjacent stages, suggesting that misclassifications are driven by the natural ambiguity of transitional developmental phases rather than random model failure. Importantly, this specific limitation has minimal clinical impact. The 3-cell stage is a highly transient state (typically lasting < 4 h), and misclassification as a late 2-cell or early 4-cell does not adversely affect the final selection priority, which focuses on Day 3 (8-cell) or Day 5 (Blastocyst) milestones [3]. Furthermore, the matrix demonstrates a high clinical safety profile, as “catastrophic” errors between disparate developmental points—such as confusing a single-cell zygote with an embryo from the “more” stage—are virtually non-existent (0.00). The “more” category, which is advanced and encompasses morulae and blastocysts, achieves a robust true positive rate of 0.96, with the only notable confusion occurring with the immediately preceding 4-cell stage (0.04). Finally, the high precision of the “Not defined” class (0.99) ensures that the system effectively segregates artifacts and non-viable samples without accidentally discarding potentially viable embryos due to false negative classification.

### 3.3. RAG-Based Explainability

We conducted a qualitative assessment of the RAG framework to evaluate its potential to overcome the transparency limitations of standard deep learning. Opaque probability scores do not offer insight into underlying biological features, they impede clinical trust. Therefore, our evaluation focused on the system’s ability to generate the clear, interpretable rationales required to mitigate liability concerns. The potential of RAG lies in its ability to convert stochastic probabilities into deterministic, rule-based logic. By grounding the model’s output in the ESHRE Istanbul Consensus, the system effectively “shows its work.” Instead of generating text based solely on statistical patterns (which can lead to hallucinations in standard Large Language Models), the RAG architecture is constrained to retrieve and synthesize only verified clinical definitions.

The application of RAG in this domain offers a dual benefit of standardization and safety. Human embryologists often use subjective or variable terminology. The RAG system forces the output to strictly adhere to international consensus language, ensuring that every report uses the exact same definitions for “fragmentation” or “symmetry” regardless of the clinic. In our experiments, a standard “off-the-shelf” LLM (like a generic Mistral-7B without RAG) relies purely on its pre-trained internal weights to generate text. This can lead to “hallucinations,” where the model invents plausible-sounding but biologically incorrect features, such as describing an “inner cell mass” in a 2-cell embryo. By contrast, our RAG system first retrieves the specific medical document for “2-cell stage.” Since this retrieved text contains no mention of an inner cell mass, the model is effectively “constrained” by the facts, preventing it from generating biologically impossible descriptions. This “guardrail” mechanism is essential for the safe deployment of generative AI in healthcare.

**Figure 13:**
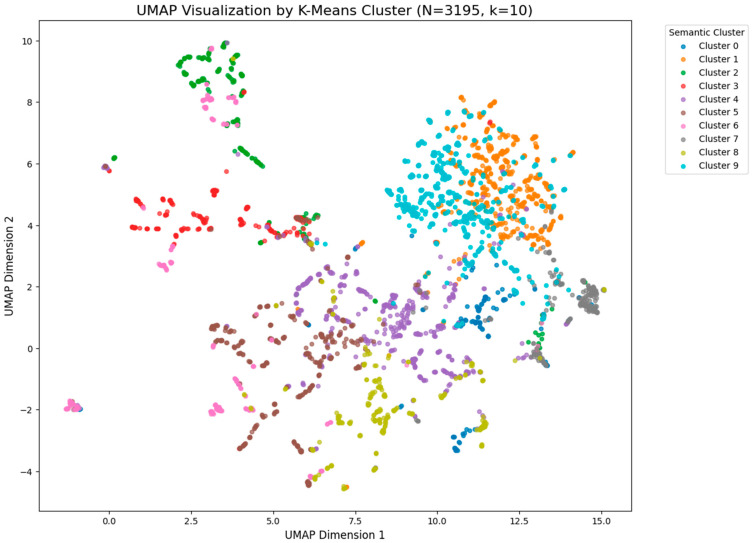
UMAP Visualization by K-Means Cluster of the semantic knowledge base. The distinct separation of clusters confirms that the database can mathematically distinguish between different developmental stages, which is a prerequisite for accurate retrieval.

We performed a qualitative validation of the semantic knowledge base to confirm the accuracy of the retrieval mechanism. As illustrated in Figure 13, the UMAP projection reveals that documents corresponding to different embryo stages naturally segregate into clearly defined groups. This semantic clustering proves that the embedding model effectively distinguishes between concepts like “2-cell” and “Blastocyst,” ensuring that the system accesses only the appropriate medical literature for a given diagnosis. Figure 13 presents a UMAP visualization of the semantic knowledge base, mapping N = 3195 vectorized text segments into a two-dimensional space clustered by K-means (k = 10). Each distinct color represents a specific semantic cluster, corresponding to different embryo developmental stages or clinical concepts defined in the medical literature. The plot reveals a clear spatial separation between these groups, forming isolated “islands” rather than an overlapping cloud, which confirms that the embedding model has successfully encoded the distinct semantic boundaries between biological categories. This structural segregation is critical for the RAG system, as it ensures that queries for a specific stage (e.g., “4-cell”) retrieve only the relevant, cluster-specific medical guidelines without interference from unrelated concepts.

**Figure 14:**
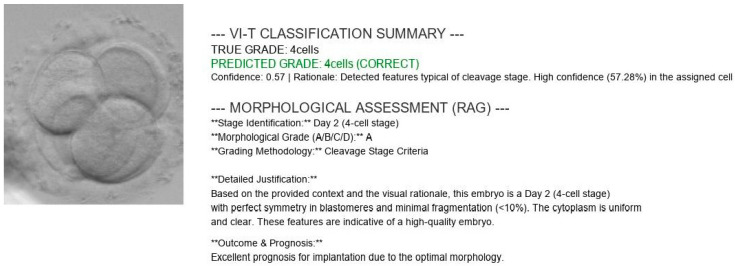
Successful Annotation Example. An image overlaid with a detailed, biologically correct text explanation generated by the Mistral-7B model on the RTX 5070 Ti.

A successful example of this pipeline in action is presented in Figure 14, where the system overlays a generated explanation onto a 4-cell embryo image. The generated report follows a structured logic:

1. Observation—Identifies the stage (“Detected features typical of cleavage stage”)
2. Confidence—States the certainty level (“High confidence in cell count”).
3. Consensus Alignment—Explicitly references the standard (“Aligns with consensus criteria for Day 2…blastomere symmetry”). This structured output demonstrates that the soft sensor successfully bridges the gap between raw pixel data and actionable clinical knowledge.

Figure 14 illustrates the final output of the soft sensor pipeline, pairing a visual snapshot of a 4-cell embryo with a comprehensive, AI-generated clinical report. The “VI-T Classification Summary” at the top verifies the diagnostic performance, explicitly matching the predicted grade of “4cells” to the ground truth with a specific confidence metric. Following this, the retrieval-augmented generation (RAG) module provides a structured “Morphological Assessment,” categorizing the embryo as Grade A based on standard cleavage stage criteria. A “Detailed Justification” section synthesizes specific biological observations, citing “perfect symmetry” and “minimal fragmentation” to explain the classification in natural language rather than simple probability scores. The report concludes with a clinical “Outcome & Prognosis,” translating the morphological data into a prediction of implantation potential, thus effectively bridging the gap between raw pixel analysis and actionable medical guidance.

## 4. Discussion

The development of the proposed “Soft Optical Sensor” addresses three critical challenges in modern reproductive medicine: the subjectivity of human grading, the inadequacy of 2D microscopy for 3D biological subjects, and the “black box” nature of AI diagnostics. By synthesizing multi-focal image fusion, transformer-based classification, and retrieval-augmented generation (RAG), this study demonstrates a viable pathway toward fully automated, objective, and transparent embryo evaluation.

Clinical Implications and Real-Practice Potential. The most immediate clinical benefit of this system is the standardization of care. Current manual grading is plagued by inter-observer variability, where the chance of an embryo being selected for transfer often depends on which embryologist is on duty. Our results show that the Swin-Tiny model achieves a consistent diagnostic accuracy of 94.11%, effectively acting as an “expert consensus” that never suffers from performance degradation. Furthermore, the integration of the RAG module allows this tool to function not just as a classifier, but as a digital assistant. By generating reports grounded in the ESHRE Istanbul Consensus, the system automates the documentation burden, potentially saving embryologists hours of administrative work per day. Ideally, this sensor would be integrated directly into time-lapse incubators, providing real-time quality control alerts without requiring the removal of embryos from their stable culture environment.

Limitations of the Research. Despite these promising results, several limitations must be acknowledged. First, the study relies on retrospective data. While the dataset is large (N = 102,308) and multi-centric, retrospective validation cannot fully replicate the pressures of a live clinical workflow where real-time decisions affect patient outcomes. Moreover, high agreement with manual morphological grading does not inherently guarantee improved live birth rates, which remains the gold standard clinical endpoint. The system currently standardizes the process of grading but requires prospective validation to prove it improves the outcome of selection. Second, the current model analyzes static images derived from Z-stacks. Although fusion recovers 3D spatial details, it misses the temporal “morphokinetic” parameters (e.g., the exact speed of cell division) which are known predictors of viability. Finally, a hardware constraint was identified in the explanation module. While visual classification runs in real-time (~7.4 ms), generating detailed text reports on standard edge hardware (CPUs) remains slow (~10 s/report). This latency represents a serious bottleneck for clinical integration, as a 10-s delay per embryo is unacceptable in high throughput laboratories. Therefore, we propose that the initial pragmatic use case must be a “tiered” workflow: offering real-time visual classification for immediate triage, while reserving the computationally expensive RAG explanations only for selected embryos (e.g., top candidates for transfer or ambiguous ‘tiebreaker’ cases). This selective application ensures that the 10-s latency does not impede the high-throughput screening of the full dataset in a live clinical environment

Future Work and Research Directions Future research will focus on overcoming these barriers. The next logical step is the development of a Video Swin Transformer capable of ingesting continuous 4D data (3D space + Time). This would allow the system to assess developmental trajectories, not just static states. Additionally, we aim to optimize the RAG component for edge deployment by investigating Neural Processing Units (NPUs) and highly quantized Small Language Models (SLMs). Specifically, we propose investigating 4-bit or 8-bit model quantization techniques to significantly reduce the memory footprint and computational load of the text generation module. Furthermore, deploying the system on dedicated edge AI hardware (e.g., NVIDIA Jetson Orin or specialized NPUs) could bridge the gap between the current 10-s latency and true real-time performance. Reducing text generation latency to under 1 s would enable a truly autonomous device. Consequently, a prospective randomized controlled trial (RCT) is not just a future option but a mandatory requirement. We are currently designing a multi-centric trial to definitively compare pregnancy rates between embryos selected by the Soft Sensor versus standard manual grading. To prove the RAG model works correctly, we will run a “blind” comparison test. Senior embryologists will grade reports written by the RAG alongside reports written by human experts, without knowing which is which. They will score the reports on a 1 to 5 scale based on accuracy, usefulness, and whether they follow ESHRE guidelines. We will then compare these scores to statistically prove that the RAG is just as reliable as a human expert.

### The Regularization Effect in Deep Learning Fusion

A counter-intuitive finding of this study is that the deep learning models (UNet-SC and AFU-Net) achieved higher average gradient scores than the Laplacian Pyramid baseline they were trained to replicate (Figure 7). This result warrants specific technical explanation given that the models were optimized using MSE. We hypothesize that this “sharpening” effect is a result of the implicit regularization inherent in convolutional neural networks. The classical Laplacian Pyramid method operates on a strict pixel-wise maximization rule, selecting coefficients with the highest local energy. While this preserves detail, it also preserves high-frequency sensor noise and introduces “soft” halo artifacts around high-contrast transitions, such as the zona pellucida. In contrast, the U-Net architecture does not process pixels in isolation but learn hierarchical feature representations. During training, the network effectively functions as a denoising autoencoder; it learns to reconstruct the consistent biological structures (e.g., cell membranes, cleavage furrows) which represent the dominant signal, while failing to replicate the stochastic noise and fusion artifacts present in the Laplacian “Ground Truth.” By filtering out these inconsistencies, the UNet-SC produces a “cleaner” image where gradients are defined by biological boundaries rather than optical noise. This results in the observed higher average gradient scores and superior visual edge definition, suggesting that the AI model has successfully generalized the logic of fusion rather than merely memorizing the pixels of the baseline.

## 5. Conclusions

This study presents a completed design for a “Soft Optical Sensor” dedicated to human embryo quality evaluation. By moving beyond traditional single-plane microscopy, we have demonstrated that fusing multi-focal Z-stack images significantly enhances diagnostic precision. Specifically, our method increased classification accuracy by 9.43% compared to standard approaches, achieving a final accuracy of 94.11%.

Crucially, the visual classification component operates in real time on standard hardware, making it practical for rapid screening. While the explainability module introduces latency, its targeted application allows the system to act as a transparent partner to the embryologist rather than a mysterious black box. This technology offers a scalable, standardized solution to reduce human error in morphological assessment. However, its ultimate utility in improving live birth rates must be confirmed through rigorous prospective clinical trials.

## Figures and Tables

**Table 1:** Comparative performance of the Soft Sensor.

Model Architecture	Test Accuracy	Inference FLOPs (G)	Latency (ms)	Parameters (M)	F1-Score (3-Cells)	F1-Score (Macro)
ViT-Base	0.965	16.87	6.86	85.80	0.831	0.956
Swin-Tiny (Edge Selected)	0.941	4.51	7.37	27.52	0.731	0.928
ConvNeXt-Tiny	0.914	1.46	3.02	27.82	0.637	0.894

**Table 2:** Comparative performance of the Soft Sensor Swin tiny (fused and just one focal plane).

Input Data Modality	Accuracy	F1-Score (Macro)	Error Rate
Single-plane (FP_0_)	84.68%	0.823	15.32%
Multi-focus fused using (UNET-SC)	94.11%	0.928	5.89%

## Data Availability

The data presented in this study are available on request from the corresponding author.

## Abbreviations

The following abbreviations are used in this manuscript:

IVF	In Vitro Fertilization
RAG	Retrieval-Augmented Generation
ViT	Vision Transformer
CNN	Convolutional Neural Network
ROI	Region of Interest
MSE	Mean Squared Error
AG	Average Gradient
ESHRE	European Society of Human Reproduction and Embryology
FP	Focal Plane
GPU	Graphics Processing Unit
UMAP	Uniform Manifold Approximation and Projection
AI	Artificial Intelligence
